# A mixed-methods approach to understanding water use and water infrastructure in a schistosomiasis-endemic community: case study of Asamama, Ghana

**DOI:** 10.1186/s12889-016-2976-2

**Published:** 2016-04-14

**Authors:** Karen Claire Kosinski, Alexandra V. Kulinkina, Akua Frimpomaa Atakora Abrah, Michael N. Adjei, Kara Marie Breen, Hafsa Myedah Chaudhry, Paul E. Nevin, Suzanne H. Warner, Shalini Ahuja Tendulkar

**Affiliations:** Department of Community Health, Tufts University, 574 Boston Avenue, Medford, Massachusetts 02155 USA; Department of Civil and Environmental Engineering, Tufts University, Medford, Massachusetts 02155 USA; Annapolis, Maryland 21401 USA; P.O. Box 399, Teshie-Accra, Ghana; Springfield, Massachusetts 01129 USA; Mayo Medical School, Rochester, Minnesota 55905 USA; Department of Global Health, University of Washington, Seattle, Washington 98112 USA; Somerville, Massachusetts 02155 USA

**Keywords:** Water infrastructure, River, Mixed-methods, Borehole, Improved water source, Surface water, *Schistosoma haematobium*, Urogenital schistosomiasis

## Abstract

**Background:**

Surface water contaminated with human waste may transmit urogenital schistosomiasis (UGS). Water-related activities that allow skin exposure place people at risk, but public health practitioners know little about why some communities with access to improved water infrastructure have substantial surface water contact with infectious water bodies. Community-based mixed-methods research can provide critical information about water use and water infrastructure improvements.

**Methods:**

Our mixed-methods study assessed the context of water use in a rural community endemic for schistosomiasis.

**Results:**

Eighty-seven (35.2 %) households reported using river water but not borehole water; 26 (10.5 %) reported using borehole water but not river water; and 133 (53.8 %) households reported using both water sources. All households are within 1 km of borehole wells, but tested water quality was poor in most wells. Schistosomiasis is perceived by study households (89.3 %) to be a widespread problem in the community, but perceived schistosomiasis risk fails to deter households from river water usage. Hematuria prevalence among schoolchildren does not differ by household water use preference. Focus group data provides context for water preferences. Demand for improvements to water infrastructure was a persistent theme; however, roles and responsibilities with respect to addressing community water and health concerns are ill-defined.

**Conclusions:**

Collectively, our study illustrates how complex attitudes towards water resources can affect which methods will be appropriate to address schistosomiasis.

## Background

Schistosomiasis is a Neglected Tropical Disease (NTD) that is poorly controlled in many communities. The disease caused the loss of 3.13 (95 % CI: 1.70–6.26) million disability adjusted life years according to the 2010 Global Burden of Disease study, but estimates of morbidity and mortality due to schistosomiasis remain controversial [9]. Urogenital schistosomiasis (UGS), which is the most common form of schistosomiasis, is caused by *Schistosoma haematobium* and is transmitted via skin contact with surface water containing cercariae. Water-related activities that expose skin to infectious water bodies place people at risk of UGS [8, 12]. Generally speaking, the main reasons for surface water contact are play by children, occupational contact, and water use for domestic purposes [6, 12], but these factors vary by location.

Recently, Mwanga and Lwambo [14] recommended that integrated schistosomiasis control strategies include assessments of knowledge, attitudes, and practices (KAP), as well as direct observations of behavior. Specifically, it is important to understand the underlying reasons for surface water contact and water use in communities where improved water infrastructure is present [7, 11, 14, 17]. Community-based field research can yield this information, providing a comprehensive understanding of transmission drivers [5–7, 15]. In particular, social science approaches to studying drivers of water-related diseases and water preferences have been recommended by a number of researchers [1, 3, 4, 7, 14, 17]. The information collected via this approach can be used to ultimately inform targeted integrated control strategies.

In 2009, believing that schistosomiasis was a community-wide health concern, members of the Council of Elders in Asamama, Ghana (pop. 2,117 in 2000, Ghana Statistical Service) invited several of the study authors to assess UGS among schoolchildren and to recommend potential control strategies. Children were screened and praziquantel was offered in both 2009 and 2010 in collaboration with Ghana Health Service (GHS). We found high UGS prevalence levels in both 2009 (48 % for girls, 50.4 % for boys) and 2010 (22.4 % for girls and 17.2 % for boys) (Kosinski, unpublished data), but data was lacking about infection drivers that were causing UGS prevalence levels to be substantially higher in Asamama compared with nearby communities. A mixed-methods, community-based study to understand the water resources context of Asamama was thus designed and conducted in 2012.

Our study objectives were the following: 1) to explore relationships among community members’ water use preferences and practices, measured water quality, and water access in Asamama; and 2) to determine whether schistosomiasis is likely to be addressed through improvements in water infrastructure. To achieve these objectives, a combination of quantitative and qualitative data was collected and explored at multiple levels of analysis (community, household, and individual level); the data sets were triangulated to improve overall confidence in qualitative results and to place the quantitative results in context.

## Methods

### Study design

A mixed-methods, community-based study was designed to provide a deeper understanding of the context of river and borehole use in a schistosomiasis-endemic community. We collected data (described below) to determine whether boreholes are available, used, preferred, maintained, and likely to provide good quality water. It is biologically implausible that borehole water would transmit schistosomiasis, while the use of contaminated surface water in endemic areas is known to be a risk factor for *S. haematobium* infection [16]; the present study was not intended to study either of these relationships. Additionally, rainwater use was outside the scope of this study, but is being assessed in detail as part of a separate study. The sampling frame for data collection, the 5 data sets, the total population sizes from which each sample was drawn, and the sample sizes for the data sets are all shown in Table 1. These data sets are described in detail below. All study activities were conducted in June 2012.

**Table 1:** Description of data sets

Data set	Data level	Data description	Sample size (n)	Sampling frame	Data use
1	Individual	Hematuria via dipstick; name; age; grade; school	260 boys	442 boys enrolled in school (3–19 years)	Data set 1 was used to describe UGS prevalence at the community level. It was also matched with data sets 2, 3, and 4 and used to determine whether infected children had poorer access to boreholes and/or better access to the river.
			255 girls	368 girls enrolled in school (3–19 years)	
2	Household	GPS coordinates of household	394 households	395 households	Household data (data set 2) was matched to the infection status of children (data set 1). Household data (data set 2) was also matched with borehole and river data (data sets 3 and 4) to assess household access to water sources.
		Demographic info. for children in household	394 households		
		Household water use preferences	247 households		
		Household attitudes re. UGS	206 households		
3	Borehole	GPS coordinates; turbidity; *E. coli*; total coliforms; O&M observations	8 boreholes	8 boreholes	Borehole data (data set 3) was matched with data sets 1 and 2 to assess access by children and households.
4	River Access Point	GPS coordinates	9 access points	9 river access points	River data (data set 4) was used with both household data (data set 2) and infection data (data set 1).
5	Community	Notes from focus group discussions	13 focus groups	adults and older teenagers (~1,200)	Data from all focus groups was assessed at the community level.

### Data set 1: Urogenital schistosomiasis screening among children in asamama

In Asamama, a total of 368 girls and 442 boys between the ages of three and 19 were enrolled in school in June 2012; they were all eligible to participate in the UGS screening portion of the study. These potential participants were informed about the study at school assemblies. The acting community head and school heads also communicated with parents and community members about the study.

Participants who enrolled in the study provided a single urine sample on one of three screening days: 6/21/2012, 6/22/2012, or 6/25/2012. Schoolchildren were asked to provide a sample between 10 and 50 mL between 10:00 and 14:00 h. All urine samples were tested for hematuria via a semi-quantitative dipstick test (U-11 Urinalysis Reagent Strips, Mindray Co. Ltd., China). Dipstick results were recorded as semi-quantitative scores and then reduced to binary data (presence/absence).

### Data set 2: Household data

There are a total of 395 houses in Asamama; the study team visited all 395 households and 394 both gave permission to collect global positioning system (GPS) coordinates and provided the name, age, grade, and school attended for schoolchildren who lived in the house. Demographic data about the schoolchildren was collected so that the house locations (Data Set 2) of children could be matched with infection data for the same children (Data Set 1).

Of the 394 households who participated in the study, a convenience sample of 247 was asked about water source preferences. Of these 247 households, 206 were also asked about whether UGS was perceived to be a health problem in the community. Handheld GPS units (Garmin GPS 72H Portable Navigator, Garmin, Ltd.) were used to collect latitude and longitude points and geographic information systems (GIS) data layers were created in ArcGIS (Esri, Version 10.1). Satellite imagery (2006 image) was used to digitize minor features such as roads; imagery was first georectified (World Geodetic System 1984, 30 N) and then features were manually created.

### Data set 3: Borehole locations and water quality

We mapped all eight boreholes in Asamama (latitude and longitude) with the same handheld GPS units that we used throughout the study. Data layers with information about borehole locations were created in ArcGIS. To quantitatively assess borehole water quality, we collected a single grab sample from each functional borehole and tested it. Total coliforms and *E. coli* were quantified via standard field test methods (filtration and incubation with m-ColiBlue24 media manufactured by Millipore). Samples were collected in pre-washed and dried 125-ml polypropylene bottles, transported on ice to the processing location, and plated within 6 h. Coliform and *E. coli* colonies were counted after a 24-h incubation period at 35 °C. Sample turbidity was measured using a portable turbidimeter (HACH 2100P). We also recorded relevant observations such as low flow rate, poor drainage in the vicinity of the borehole, visible particulate matter, etc.

### Data set 4: River access points

All regularly used river access points in the community were visited and mapped with one of two community members who acted as guides. The same handheld GPS units were used throughout the study to collect latitude and longitude points; data layers were created in ArcGIS.

### Data set 5: Focus group discussions

Teenage and adult participants were recruited for 13 focus groups (Table 2) by explaining the study verbally in public spaces such as the main market, at shops, and at open-air hair salons. Focus group discussions were all conducted on 20 June, 2012 and lasted approximately 45 min each. Participants were purposively sampled by seeking heterogeneity in age, gender, and geographic location, but women were oversampled given the role commonly played by women with respect to water collection, water storage, and water use. No attempt was made to connect focus group participants with any of the children in the study, although it is very likely that some participants were family members of children who were screened for hematuria. Focus group data was collected to identify community perceptions of and popular discourse on water resources, water infrastructure, and UGS. Data from the focus group discussions provided context for how and why the river and improved water sources were or were not being used.

**Table 2:** Focus groups compositions; D1-D4 represents each discussion leader; T1-T2 represents the two translators

Group #	Group composition	Focus group setting	Discussion leaders	Translators
1	~5 women, 2 men, all between 20 and 40 years old	Private home	D1	T1
2	4 young to middle-aged women	Small shop	D1	T1
3	2 middle-aged women	Small shop	D2	T1
4	2 middle-aged women	Private home	D2	T1
5	6 women ranging from late teens to 40s	Hair salon	D2	T1
6	2 women in their 30s or 40s and 1 man in his 30s	Small shop	D2	T1
7	3 women in their 20 and 30s	Hair salon	D3	T2
8	3 middle-aged women	Central market	D3	T2
9	3 women in their 30 and 40s and 1 man in his 30s	Central market	D3	T2
10	4 males around 17 to 20 years old	Near main road	D3	T2
11	2 middle-aged women	Small shop	D3	T2
12	3 women in their 20 and 30s, 1 woman in her 50s	Small shop	D4	T2
13	2 women in their 30, 2 boys in their teens, 2 men in their 40s	Private home	D4	T2

Four focus group discussion leaders and two translators worked with the focus groups (Table 2); each group had a note-taker. Discussions were semi-structured and all focus group leaders used the same open-ended questions and probes. Questions related to the following topics: community priorities; community health concerns; and local perceptions of water infrastructure, surface water, and UGS. Discussions were recorded in note format.

### Data analysis

The prevalence of hematuria for boys and girls in Asamama was assessed using Data Set 1. To explore the relationships among infection status, house location, household water source, and household perceptions of UGS, Data Sets 1 and 2 were manually matched using demographic information. Discrepancies in reported names, ages, and schools of children made it impossible to match all children in the two data sets. The 394 households in Asamama that agreed to participate in the study reported a total of 784 children and it was possible to assign house locations to 367 children who were tested for hematuria; in total, 793 children were enrolled in school in Asamama and of these, 515 were screened for hematuria. Thus, 71.3 % (*n* = 367/515) of all children who were screened were matched with their household locations. After the matching process, Data Sets 1, 2, 3 and 4 were analyzed together in ArcGIS using proximity tools. A buffer analysis was conducted to determine whether access to improved (boreholes) and unimproved (river) water sources differs among infected and uninfected children in Asamama. This analysis involved calculating the percentages of infected and uninfected children living within varying buffer distances of boreholes and river access points. The ‘near’ analysis tool in ArcGIS was also used to calculate distances from the households to the nearest water features. Subsequently, a Welch Two Sample t-test was conducted in R software (version 3.1) to test whether or not there were significant differences in distances from boreholes and river access points among households that reported different water source preferences in Data Set 2. Data Set 2 was also used to assess correlations among perceptions of UGS as a health concern and water source preferences.

For analysis of focus group data (data set 5), we fully transcribed our notes and imported them into Dedoose for analysis (2011 SocioCultural Research Consultants, LLC). Two senior team members deductively coded the transcripts of the focus group discussion notes using a start list of a priori codes. As additional themes inductively emerged from the data, they were added to the codebook and confirmed for consistency between coders.

### Ethics, consent, and permissions

Officials in Atiwa District, Eastern Region, Ghana, provided written permission to conduct this study. The Atiwa District Chief Executive, the Head of the Atiwa District Ghana Health Services, the Atiwa District Superintendent of Schools, the acting head of the community, and the acting head of each school all provided written permission for the study. Adult participants ≥ 18 years) provided verbal informed consent while verbal assent was obtained from child participants (<18 years). Finally, nurses and/or community health workers from GHS offered praziquantel to all study participants who tested positive and to any child who wished to be treated, regardless of participation in the study. The Social, Behavioral, and Educational Research institutional review board of Tufts University and the institutional review board of the Noguchi Memorial Institute for Medical Research in Accra, Ghana approved this study.

## Results

### UGS prevalence

Table 3 shows the prevalence of hematuria by age for both boys (*n* = 260) and girls (*n* = 255). There is no statistically significant difference between the prevalence of infection in boys and girls (29.8 % for girls, 34.2 % for boys, *p* > 0.05). The mean age of infected girls was 10.0 years versus 9.76 years for infected boys (*p* > 0.05).

**Table 3:** Age and sex of study participants who tested positive for hematuria

	Girls			Boys		
Age	# Positive	% Positive	Total	# Positive	% Positive	Total
3	0	0	7	0	0	7
4	3	27	11	4	27	15
5	1	6	16	8	40	20
6	7	33	21	8	42	19
7	8	40	20	3	25	12
8	15	63	24	9	36	25
9	4	33	12	7	35	20
10	8	33	24	11	65	17
11	4	27	15	8	50	16
12	8	25	32	11	37	30
13	4	19	21	9	35	26
14	3	17	18	4	19	21
15	5	24	21	6	33	18
16	4	40	10	1	25	4
17	1	50	2	0	0	2
18	0	---	0	0	---	6
19	1	100	1	0	0	2
Total	76	29.8	255	89	34.2	260

### Water use, water quality, and UGS

**Fig. 1:**
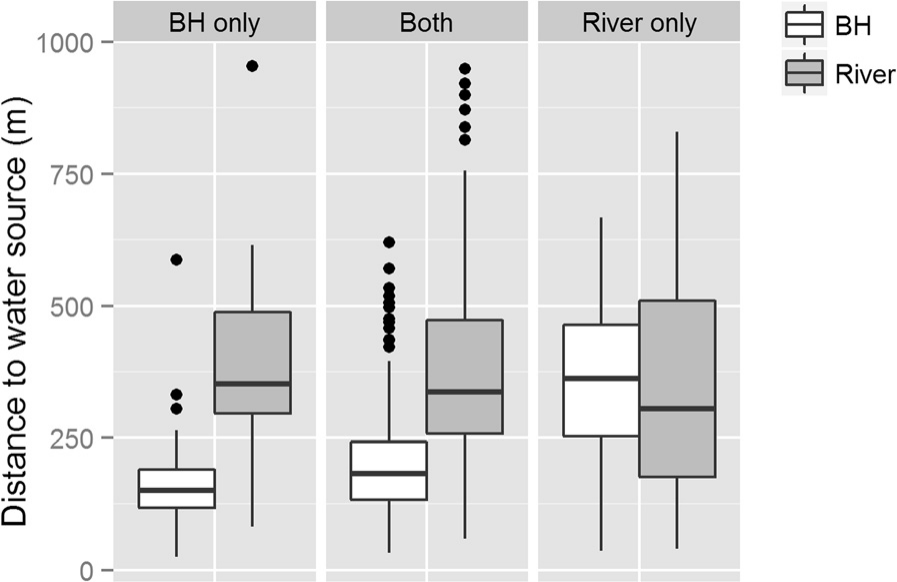
Boxplots showing distance from households to boreholes and river access points in Asamama

Contact with river water is the only means of *S. haematobium* transmission within Asamama; thus, a major goal of the study was to understand the reasons for river contact, particularly given that eight boreholes exist in this town. 87 (35.2 %) households reported using river water but not borehole water; 26 (10.5 %) households use borehole water but do not use river water; and 133 (53.8 %) households use both river water and borehole water. Distances to water sources (functional borehole vs. river access point) were compared within these groups; at the time of the study, boreholes 1, 3, 4, 7, and 8 were functional (Table 4). For households that use only boreholes and households that use both boreholes and river water, boreholes are significantly closer to people’s homes than are river access points (*p* < 0.0001) (Fig. 1). For households that use only the river as a source of domestic water, there is no difference in the distance between river access points and people’s homes and the distance from boreholes to homes (*p* = 0.9625).

**Table 4:** Results of water quality tests of public boreholes in Asamama

Borehole ID	Turbidity	*E. coli*	Total Coliform	Notes
	(NTU)	(col/100 mL)	(col/100 mL)	
1	15	0	38	Near trash dump, particulate matter observed
2	--	--	--	Not functional
3	2	0	4	
4	12	2	59	Poor structural condition
5	--	--	--	Not functional
6	1	0	4	Low flow rate
7	25	0	2	Near trash dump, particulate matter observed
8	3	1	101	Poor drainage

**Fig. 2:**
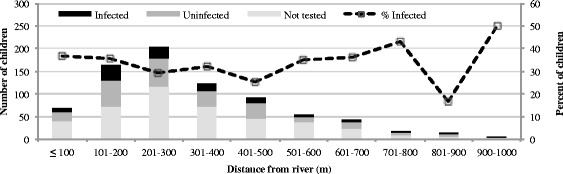
Children living within a given distance of a river access point, grouped by hematuria status

To determine whether distance to water sources correlates with the infection status of school-aged children, distances to a functional borehole or to a river access point were plotted against infection status (Fig. 2). Approximately half of the children in the various distance bins were screened and half were not, indicating an unbiased sampling procedure with respect to distance from a water source. Hematuria prevalence does not differ by borehole or river access, which is clear from the line indicating percent infected on the secondary axis; percent infected remains relatively constant across the various distances, except when the sample size is very small.

**Fig. 3:**
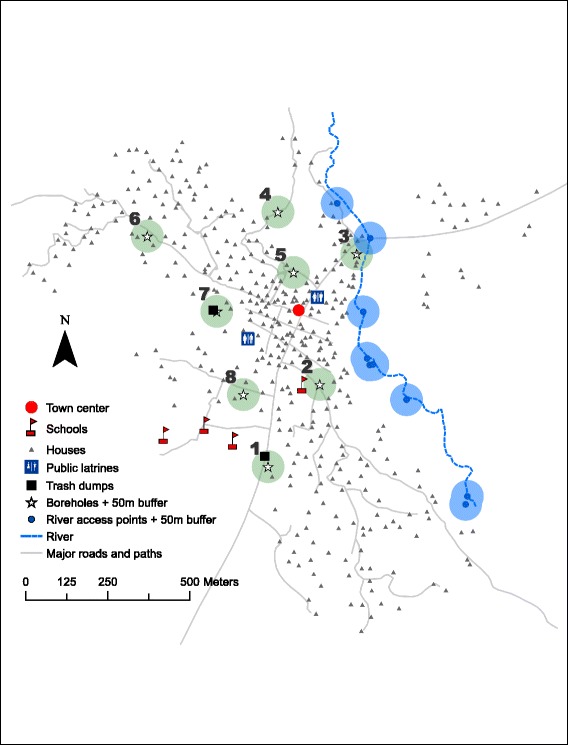
Spatial layout of Asamama; boreholes are numbered for reference in the text

The spatial layout of Asamama (Fig. 3) shows the access points on the Abresu River and the locations of all eight boreholes. All households are within 1 km of a functional borehole. Boreholes in Fig. 3 are numbered to match with information in Table 4. Boreholes 2 and 5 were not sampled because they were not functional at the time of data collection. Boreholes 1 and 7 are located directly beside rubbish dumps. Boreholes 4, 6 and 8 had structural deficiencies or problems with operations and maintenance (O&M). High turbidity levels (12–25 NTU) were found in 3 of 6 boreholes tested. Relatively high total coliform levels (38–101 cfu/100 mL) were observed in boreholes 1, 4, and 8. Boreholes 4 and 8 had low levels of *E. coli* contamination (1–2 colonies/100 mL). Only borehole 3 was found to have neither complaints from community members nor observed water quality problems.

### UGS perceptions & water preferences

We asked 206 (52.3 %) households whether they believed that UGS was a problem in the community; all 206 provided a response. 89.3 % of households said that in their opinion, UGS is a problem. There were no statistically significant differences between perceptions of UGS as a problem when comparing households that use only borehole water with either households that use exclusively river water or with households that use both types of water sources (Range: 84.9 to 100 %; *p* > 0.05). Focus group data provided additional context.

### Themes from focus group discussions

**Fig. 4:**
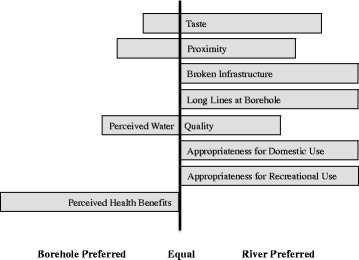
Detailed Legend: Reasons for preferring borehole water or river water for domestic needs; given the qualitative nature of the data and the varying compositions of focus groups, the data are presented to reflect general trends rather than quantitative values. Bars are all the same length; the horizontal location of the bar refers to people’s preferences (either towards river or towards borehole)

We categorized focus group discussion topics into themes. The reasons for why the river is used and why boreholes are not used were frequently discussed together. People were generally aware of the dangers of using river water; for example, they often mentioned UGS and diarrheal disease (specifically cholera and typhoid) and they recognized that sewage and agricultural runoff contaminate the river, yet most people still reported preferring the river for a variety of reasons (Fig. 4). The primary reasons for this preference were taste, proximity, broken boreholes, and the perceived appropriateness of river water for domestic and recreational use.

In terms of the appropriateness of river water and borehole water, people use relatively large quantities of water for tasks such as bathing and washing clothes. It is faster and easier to collect large quantities of river water, as compared with borehole water, particularly if there is a queue at the nearest borehole, if the flow rate is low, or if the nearest borehole is broken. In addition, participants in Focus Group 13 explained two of the specific concerns people have about using borehole water for domestic purposes: borehole water does not form lather well with soap, and when borehole water is used to make palm oil, not enough oil separates. These reasons are clearly non-exhaustive, but these two complaints were common throughout the community; in particular, soap failing to lather is considered a problem by community members and causes them to use river water for laundry, bathing, and dish washing.

The river is widely perceived to be appropriate for swimming for both adults and children. There are separate areas of the river that are used by the two groups, and both children and adults use the river regularly for recreation. In some focus groups, participants mentioned the idea of UGS risk and the connection between swimming and infection. Some focus groups recommended the creation of alternative options for recreation among children (ex. a community center, a swimming pool, etc.).

Responses were split on water quality. Some people reported that they and their ancestors have always used the river, so therefore river water is fine to use; the rationale was that the ancestors did not become sick or die from using river water. However, many people discussed the fact that town gutters drain into the river and the river water quality has been degraded over time. Knowledge of these water quality problems and changes in quality over time do not prevent people from using the river since water quality in boreholes is also perceived to be poor. On many occasions, people saw an oily sheen on water collected from boreholes. They also reported that particulate matter and pieces of rust were seen in the bottom of plastic buckets of settled borehole water. Lastly, people complained that two of the functioning boreholes are near the trash dump so they do not like to use them.

The theme of “responsibility” was pervasive in focus group discussions. People discussed responsibility with respect to maintaining infrastructure and addressing UGS. Most focus groups discussed a perceived lack of leadership and a lack of action by the elders in addressing community problems. People tended to feel that it is the responsibility of the *leaders* to address community problems; the belief that community *members* should accept some of the responsibility is rare: only one person in one focus group discussion mentioned it. The same is true of boreholes; while people complained that community leaders and the relevant town water committee do not fix them, end users do not feel responsible for broken boreholes, either.

## Discussion

Approximately two thirds of all households report regularly using river water. Distance plays a significant role in determining whether households will use only borehole water, a combination of sources, or only river water for their domestic needs. Improving access to borehole water by drilling new wells or installing a pipe distribution system with standpipes might increase the use of improved water sources. However, this type of intervention is unlikely to result in decreased recreational water contact, which could be a major driver of UGS; the role of recreational water contact in maintaining transmission should be explicitly studied. A household’s geographic access to boreholes or a river did not appear to impact UGS prevalence among children, but this could be due to the relatively close proximity of the river to most households. To address recreational contact, it might be feasible to construct water-based recreation facilities in Asamama and in similar towns. For example, a swimming pool ([13] and 2012; [10]), shallow wading pool, or water sprinklers could meet recreational needs for children with no risk of UGS, but these options all demand substantial maintenance and may be too costly for some communities.

Since all boreholes in this community were within 1 km of a functional borehole, strictly in terms of *distance* and the Millennium Development Goals, people in this community have access to an improved water source [20]. However, the Millennium Development Goals also stipulate that access to improved water means the water meets microbial, chemical, and physical standards, as established by either the Ghanaian government or by the World Health Organization [20]. Our data indicate that there are a number of serious water quality problems with most boreholes in the community, which may partially explain why community members do not strongly prefer borehole water to river water. For example, the recommended turbidity for drinking water is typically less than 1 NTU but for some water sources, a target of 5 NTU is used [19]; all boreholes in Asamama had an NTU reading of 1 or more, and three were above 5 NTU. Second, *E. coli* should not be detectable at all in a 100 mL drinking water sample [19], but we found low levels of *E. coli* contamination (1–2 colonies/100 mL) in two of the boreholes.

New boreholes will not address the fact that most boreholes in this community had structural problems or poor water quality. Only one of the eight boreholes had neither complaints from community members nor observed water quality problems. However, this borehole (#3) is very close to a river access point, which could mean that it is under-utilized, particularly if there is a queue to use it or if patrons are expected to pay for use or for repairs. If efforts are made to improve access to borehole water, research should first be conducted to understand the drivers of good and poor borehole O&M to ensure that water quality and quantity are consistently acceptable. We also recommend that organizations that donate boreholes to rural communities plan carefully for O&M throughout the project’s lifetime [2, 18]. More work needs to be done to understand the causes of particulate matter, oily sheen, rust, and other contaminants in the boreholes and to regularly carry out repairs. Outreach should also be done to explain naturally-occurring groundwater quality problems to the community (ex. water hardness). The confusion about roles and responsibilities with respect to addressing infrastructure problems needs to be addressed as well, as this confusion contributes to poor borehole maintenance.

To address water quality problems, focus group discussion data suggests that people prefer individual versus community-based interventions. People would rather receive point-of-use water treatment options to treat their own water versus receiving additional boreholes where water quality may be poor and proper maintenance may not occur. Focus group discussion data also suggests that if public water infrastructure were substantially improved or newly implemented, people would prefer to pay on a ‘per-use’ basis rather than via a monthly fee. Careful consideration should be given to the payment mechanisms that are used to generate funds for borehole repair; additional studies should be conducted to determine which payment mechanisms will result in steady revenue streams that are sufficient to cover O&M.

In Asamama, water for domestic use is manually carried to homes. Women, teenagers, and children most frequently carry out this task. Focus group discussion participants generally agreed that proximity to a borehole or the river is a major determinant of use. In Asamama, although there is widespread recognition of UGS as a health concern, there is no evidence from focus group discussions that people chose a water source based mainly on the perceived health benefits. Moreover, households that use only borehole water were not more likely than households reporting only river use to perceive that UGS is a problem in the community. This suggests that UGS as a health concern is not a major driving factor when households make decisions about water sources. However, the lack of statistical significance could be real, or it could be due to a relatively small sample size or to difficulty gaining access to borehole water in certain regions of the community, which would mean that water use is not primarily determined by perceptions of UGS risk. Additional research is needed to understand the primary drivers of water source selection in rural Ghanaian communities.

This study had several limitations. The lack of a statistically significant difference in water source preferences between groups with varying perceptions of UGS as a problem could suggest a gap in knowledge about how UGS is transmitted and what types of precautionary measures might be taken to protect against infection, but it could also reflect a general dissatisfaction with borehole water in the community, or could be due to a small sample size. These are important areas for future research. Focus group discussion data is useful for placing study findings in context, but in future studies, it would be interesting to tape-record and fully transcribe all statements by participants. Finally, a convenience sample was used to ask households about water source preferences. While a large percentage of all households were asked this question, it would be interesting to compare our results with the results of a randomly selected sample of households.

## Conclusions

New boreholes and better O&M of existing infrastructure could increase the use of improved water sources, but these enhancements are unlikely to reduce recreational water contact, which is a known risk factor for UGS. There are a variety of factors, such as distance, price, and functionality, that water users weigh when selecting a water source, but risk of schistosomiasis and other health concerns do not appear to be priority considerations.

### Availability of data and materials

Due to ethical restrictions imposed by the Tufts University Institutional Review Board related to protecting participant consent and confidentiality, data are available upon request to the corresponding author.

## Abbreviations

GHS: Ghana Health Service
KAP: knowledge, attitudes, and practices
NTD: Neglected Tropical Disease
NTU: Nephelometric Turbidity Units
UGS: urogenital schistosomiasis
